# Special operations: a hidden chapter in the histories of facial surgery and human enhancement

**DOI:** 10.1136/medhum-2019-011792

**Published:** 2020-07-05

**Authors:** Roderick Bailey

**Affiliations:** Wellcome Centre for Ethics and Humanities, University of Oxford, Oxford, UK

**Keywords:** aesthetic/plastic and reconstructive/cosmetic surgery, medical ethics/bioethics, medical humanities, law, history

## Abstract

During the Second World War, Britain’s Special Operations Executive (SOE), a secret service established to encourage resistance and carry out sabotage, employed various techniques of enhancing the ability of its personnel to operate undetected in enemy territory. One of these methods was surgery. Drawing on recently declassified records, this article illuminates SOE’s reasons for commissioning this procedure, the needs and wants of those who received it, and the surgeons employed to carry it out. It also aims to underline the role of context in shaping perceptions of facial surgery, and the potential for surgery for wartime disguise to resonate with current debates about human enhancement.

## INTRODUCTION

In 1942, border guards on Fascist Italy’s frontiers received instructions from Rome to look out for an enemy agent trying to enter the country in disguise. Giovanni Di Giunta, Rome explained, was a Sicilian ex-soldier in his thirties and a clandestine agent of the British, and his reason for entering Italy was to assassinate Mussolini. An old photograph found in army records was also circulated, ‘but we inform you’, the communiqué warned, ‘that as a result of a plastic surgery operation’ Di Giunta would be ‘modified’ in the following way: his nose had been narrowed, his chin shortened and his cheekbones made less prominent, and a scar to his upper lip lengthened towards his right nostril.1 Today, the files of Britain’s Special Operations Executive (SOE) confirm that Di Giunta was indeed an ex-soldier from Sicily whom the British had prepared for a mission to kill Mussolini, and that he had received surgery to assist him.2 Those files also show that SOE subsequently switched his target to one of Mussolini’s lieutenants, Roberto Farinacci, before abruptly cancelling the mission when doubts developed about Di Giunta’s seriousness.3 His description came to the attention of the Italian authorities when a fellow agent, to whom he had disclosed his secret task before SOE scrapped it, was captured and interrogated.4

Surgery for the purpose of wartime disguise has received scant attention in print, despite extensive interest in the history of espionage and a growing body of humanities literature on facial surgery. This paucity may be explained by the handful of hitherto-published references existing largely in memoirs long out of print,5 and by the fact that corroboration, in the form of contemporary documentation, has only become accessible recently. Except for a brief piece by this author, little exists publicly to enrich the picture.6

Historians have demonstrated how studying elective and facial surgery can advance understanding of modern societies on a range of fronts: from the role of military imperatives in promoting reconstructive surgery7 to the phenomenon of ‘passing’8—the sociological concept of a person’s efforts and ability to be regarded as a member of a group different from their own—and related concepts of beauty and ugliness, links between physical appearance and mental health, and the influence and impact of consumerism.9 Drawing chiefly on SOE’s declassified records and the files of its American cousin, the Office of Strategic Services (OSS), with which SOE occasionally shared details, what follows aims to show that exploring the use of surgery for wartime disguise can be similarly instructive. Encouraged, in particular, by the recent writing of Sharrona Pearl, whose cultural study of face transplant surgery urges greater awareness of how societies think about faces,10 it seeks to present a clearer image of this surgical work and the perceived needs for it while highlighting the limits of viewing the practice through the lens of peacetime cosmetic surgery. Studies that have dominated the history of that field, such as those by Elizabeth Haiken and Sander Gilman, frame rationales for these interventions largely as psychological and emotional responses to cultural pressures and norms.11 Other writers stress the importance of biology alongside culture.12 It will be shown here that distinct dynamics also fuelled demand for facial disguise in wartime, and that examining these illuminates not only the motivation and self-perception of those who received it but also how some surgeons have conceived the justifications for cosmetic surgery, their moral and national duty in time of war, and the risks inherent in such work. As such, it engages with medical historian Thomas Schlich’s remark that emotions in history can usefully illuminate the ‘extent [to which] feelings are universal and time-independent or contingent and shaped by their environment of their time’.13

The article opens by demonstrating that this unusual surgery was in the hands, and driven by the needs, of a nation-state at war. It then looks more closely at the question of motive, arguing that, for some recipients, reasons for facial disguise were more nuanced than a simple wish to escape enemy surveillance. Attention then turns to the participating surgeons. One left an account of his involvement. Its accuracy is hard to judge, but his expressed doubts about the justification of surgery for wartime disguise encourage discussion of its ethical and legal dimensions, as well as the current pertinence of these to questions about modifying the body for war.

## ‘PERMANENT MAKE-UP’

SOE was a secret service established by the British Government in 1940 to encourage resistance and carry out sabotage. Its principal method was to dispatch trained agents into enemy territory. Most of these were not British. SOE needed volunteers who could pass convincingly in the countries in which it was trying to operate, and the most suitable recruits tended to be nationals of the countries concerned. Many still needed assistance to operate successfully, however. Special training, in the use of weapons and radio transmitters, for example, often helped. But to stay undetected in enemy territory could require other forms of aid. The authors of four memoirs would claim that this assistance included surgery. Today, declassified records confirm that this was so. These new sources also demonstrate that, from SOE’s perspective, it was a practice performed entirely in the interests of the state.

The first former agent to publicly claim to have had surgery appears to have been James Hutchison, a Scotsman who had parachuted into France in 1944. A year later, campaigning for a parliamentary seat in Glasgow, he gave journalists a few details of his wartime exploits and was not shy of mentioning the surgery done. ‘I entered a clinic and had my face permanently altered’, he was quoted as saying. ‘The bridge was removed from my nose and the tips of my ears were clipped’.14 SOE records reveal that senior officers still serving in the organisation were not pleased with these admissions (‘It is entirely vulgar of course and will encourage other heroes to publicise themselves’)15 but he seems to have been left un-censured: in later memoirs, he described how, in London, a plastic surgeon had removed the tops of his ears, reduced the size of his nose and fashioned a more prominent chin, the latter by the addition of a sliver of pelvis accessed through an appendix scar.16

By then, two other ex-agents had published accounts of receiving facial surgery. In his own memoirs, which appeared in Danish in 1950^17^ and in English in 1956, Flemming Muus, a Dane, wrote little about the precise work done but remembered ‘a painful operation, carried out under a local anaesthetic. The surgeon insisted that I should follow the procedure by holding a looking-glass in front of my face. It was not very amusing… They had to sew through my ears to keep them back after the operation, but although the doctor put seven stitches in, he did not consider it necessary to anaesthetise them’.18 Also in the 1950s, first in French,19 then in English, George Langelaan, a British army officer recruited to work for SOE in occupied France, recalled two operations in a civilian clinic in London. One had pinned back his ears and reduced the size of the lobes. The next had distorted the shape of his chin by enlarging it with a bone graft taken from his thigh.20

A fourth account mentioning surgery has appeared more recently. In a memoir published posthumously in 1998, Jean-Pierre Levy, a prominent figure in the French Resistance, recalled how, during a short trip to London in 1943 for briefings and SOE training, he had received ‘special treatment’ to remove a scar on his right cheek.21

Although all of those authors stressed the novelty of their experience, the techniques used were not new: by the 1940s, cosmetic procedures of this type were more-or-less standard practice. Indeed, while historians of surgery have described their topic as ‘an ideal field for examining the processes of technological change in medicine’ and, in the context of facial surgery, pointed often to the formative role of the First World War in pioneering methods of cosmetic reconstruction, this tale is not one of innovation, except insofar as it presents an instance of established techniques receiving new meaning.22 Moreover, SOE records make clear that this was state-sponsored surgery concerned with maximising the potential of healthy bodies to assist in the defeat of a nation’s enemies. Indeed, responsibility for recommending it lay not with SOE’s medical staff but with Section XV, SOE’s Camouflage Section. This had its principal workshops in the grounds of a mock-Tudor roadhouse off the A1 in Hertfordshire, north of London, where its staff devoted themselves mostly to concealing the tools of clandestine warfare, such as documents, radios, weapons and ammunition. The standard approach was to disguise them inside everyday objects that could be delivered into enemy territory and used, moved or stored without arousing undue suspicion. Details survive among SOE files of a wide and imaginative selection of solutions, from radio transmitters hidden in gramophones to explosives concealed within fake rocks and logs. The same line was taken with agents’ bodies. Typically, this meant providing suitable clothing that would appear unremarkable in countries where they would operate: an agent going to France, for example, might be issued with civilian clothes complete with French labels and cut according to French fashions. But it also meant assisting with measures of ‘personal disguise’, as SOE called them. This was where plastic surgery came in.23

**Figure 1:**
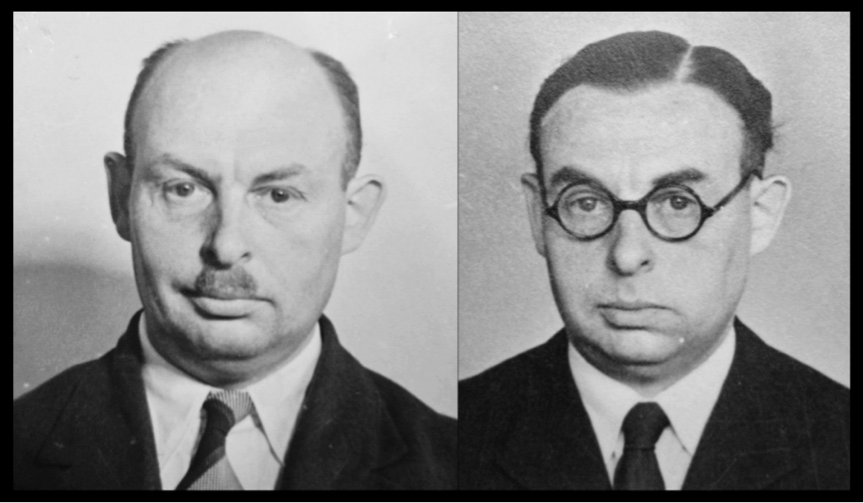
‘Semi-permanent make-up’: Charles Claser, Belgian resistance fighter, before (left) and after (right) SOE assistance with temporary disguise. TNA HS 6/47.

As an in-house history of the Camouflage Section explains, ‘personal disguise’ was divided into three categories of ‘make-up’: temporary, semi-permanent and permanent. ‘Temporary make-up’ was defined as ‘a measure of emergency camouflage’ that could be achieved with speed and ease. It covered a variety of simple techniques, some of which could be prepared before agents departed. Examples included the following: glasses; cosmetic make-up; false moustaches; gold or porcelain dental caps that could be slipped over real or false teeth; and tips on how to appear taller, shorter, richer, poorer and even lame (the latter by placing a stone in a shoe to assist with faking a limp). ‘Semi-permanent make-up’ was designed to produce an effect that was more convincing and lasted longer. Tending to require more effort, it included such measures as hair-dying, teeth-staining, eyebrow-plucking (to make eyes seem further apart), facial massage (to make skin seem younger), contact lenses (to change eye colour) and reshaping mouths and nostrils with removable pads. Some agents were fitted with wigs and toupees (Figure 1). Scars, burns and tropical ulcers could be simulated with cosmetics. For operations in Asia, skin dyes were developed to help agents blend more easily with local populations.24 OSS even heard that SOE used X-ray radiation to make men bald: ‘Complete baldness with a persistence of four to five months is effected’.25

The third category, ‘permanent make-up’, meant dental work and plastic surgery. An OSS report from 1944, written after its London representatives learnt about SOE’s work in this area, provides more detail:

Nose operations usually require about a week or ten days’ hospital care, but a month must be counted upon before all swelling has gone down and the resulting “black eyes” have disappeared. One advantage is that there are no outside scars.

Prominent ears are dealt with by pinning them back. This leaves a small inconspicuous scar where the skin joins the ear to the head. Two to three weeks should be allowed for this.

Scars that show are definite and dangerous marks of identification which should always be eliminated if possible. They can be removed surgically by a specialist without requiring hospitalization [sic]. An operation lasting two to three hours and removal of stitches after seven to ten days are all that are necessary.26

**Figure 2:**
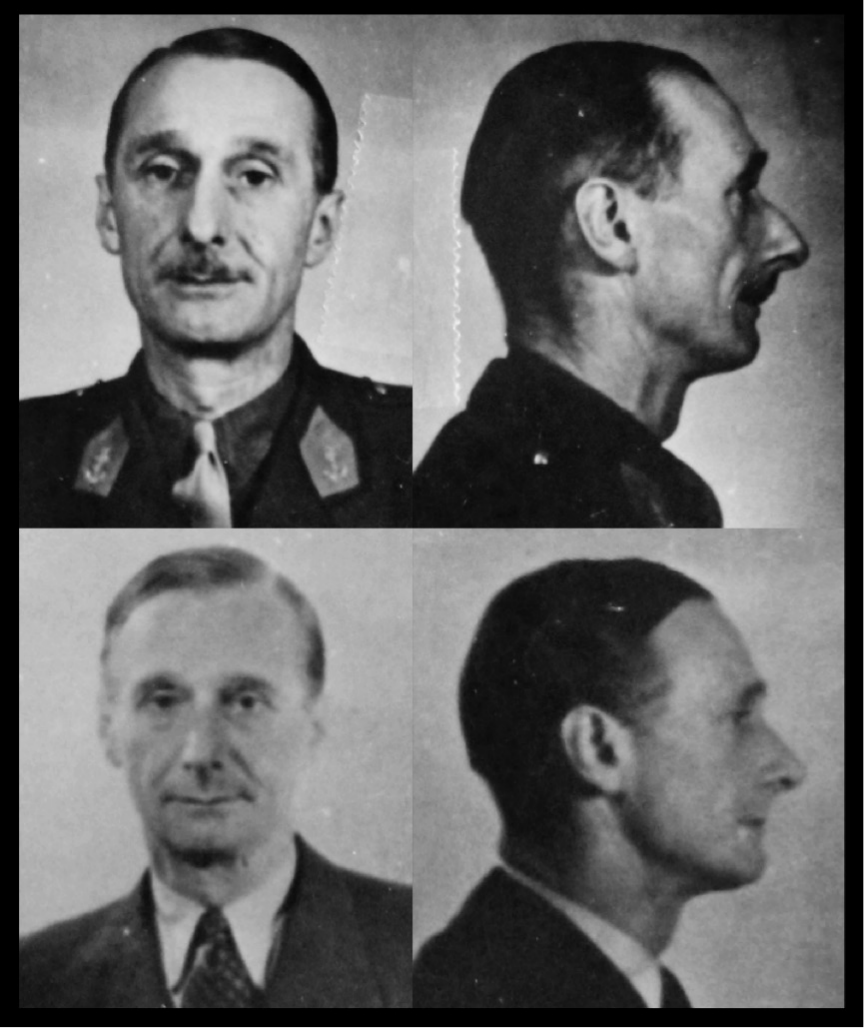
Anonymous agent photographed before (top) and after (below) surgery, showing evidence of rhinoplasty. TNA HS 7/49.

**Figure 3:**
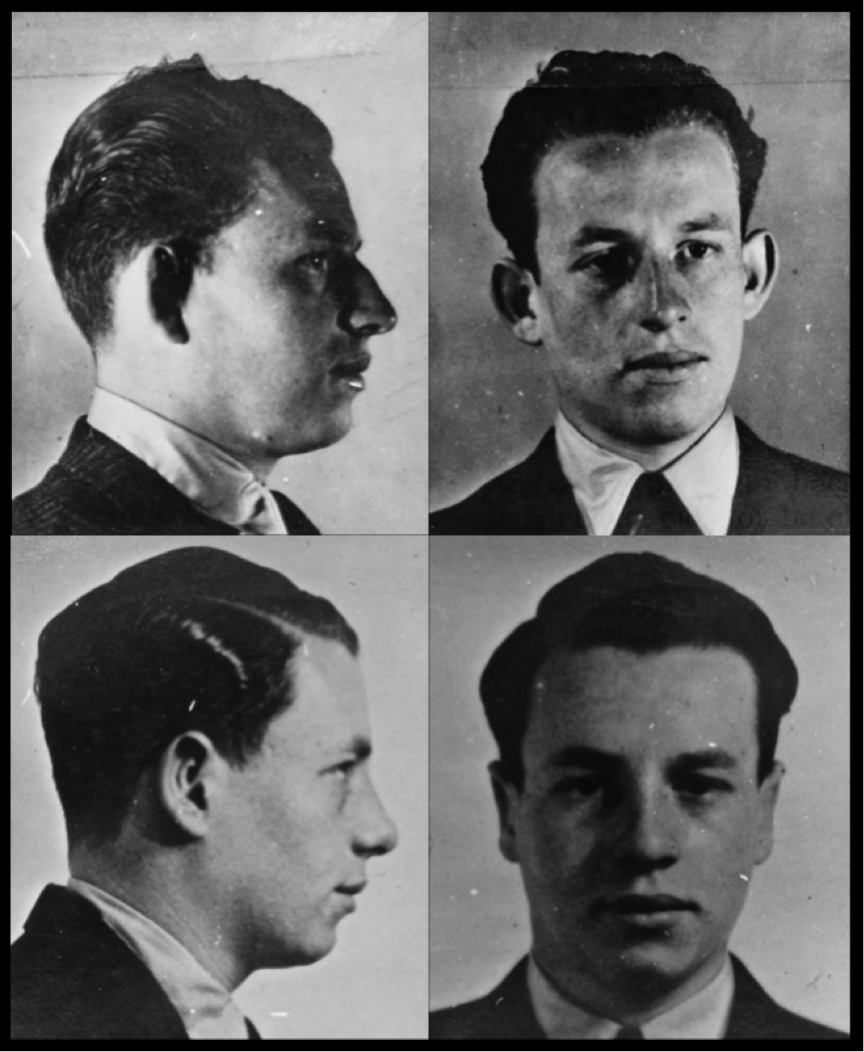
Anonymous agent photographed before (top) and after (below) surgery, showing evidence of adjustments to nose and ears. TNA HS 7/49.

Section XV’s reports of camouflage work completed or due to be done provide anonymous glimpses of more recipients of surgery: ‘Plastic operation on forehead’; ‘Surgical operation to ears’; ‘Remove or disguise scars on forehead’; ‘Surgical operation to repair broken nose’.27 Its in-house history, meanwhile, includes before-and-after photographs of three men, all unnamed, to illustrate visually the type of work done. Each ‘after’ image demonstrates evidence of rhinoplasty (surgery to the nose) (Figure 2 and Figure 3). One shows also a pair of pinned-back ears (Figure 3).28

Nowhere in SOE’s surviving files are these procedures described as health-related or concerned with making features more attractive to the eye: this surgery was about camouflage. Underlining this, OSS documents record that, in the autumn of 1944, Bill Osborne, a civilian employee whose prewar career had been in prop-work for the film industry, was responsible in SOE for arranging it. Osborne worked with Section XV’s Photographic and Makeup Department, known as Section XVc, in premises at 2–3 Trevor Square in Knightsbridge: a location better suited than Hertfordshire for liaising with the London-based country sections whose agents needed help.29 ‘Mr. Osborne studies the agent being outfitted after receiving the cover story and location of the mission, advises with the [country] desks, and then supervises any alteration required’, OSS recorded. ‘He has his own women hairdressers to handle simple “hairdos” and a professional makeup artist for color [sic] work. Any difficult hair jobs are sent to the best commercial hairdressers in London; dental work, such as replacing a British plate with one of German workmanship, to an outstanding dental surgeon… and plastic surgery… [for operations to] noses and scars, face lifting, etc., to the surgeon.’30

## SUBJECTS AND STIMULI

All agents who recorded, in print, their motives for surgery had, according to them, feared recognition by people who knew their true identities. James Hutchison, who had occupied a command role for SOE in London prior to parachuting into France, suspected that captured agents had given his description to the Germans, so he was cautious about following them into the field.31 ‘I had a large circle of friends in Denmark’, Flemming Muus recalled.32 George Langelaan, a journalist in prewar Paris, was anxious for a similar reason and especially about his ears, which he described as ‘standing right out and inquisitively facing people, as though they wanted to see as well as hear them’.33 The memoirs of the British officer who took him to the surgeon corroborate Jean-Pierre Levy’s concern that a scar made him conspicuous: ‘it was feared that his grim features were too well known to the Gestapo, who wanted him dead or alive’.34 But motives for anonymity could be more complex than this. In particular, some agents feared that physical attributes associated with race could put their lives at risk.

Ongoing study of SOE personnel files, which continue to be released, has revealed, to date, three more recipients identifiable by name. All were Jewish agents anxious that their noses conformed too much to Jewish stereotype. One of them was Guy Pevtchin, a student from Brussels, whom SOE recruited in 1943 after his escape from Belgium to Britain.35 Under consideration for a mission into Nazi Germany in the guise of a foreign labourer, Pevtchin underwent rhinoplasty in early 1945 after SOE expressed concern that agents of a ‘physically striking semitic type’ might be poorly suited to that role: ‘Germans do not employ Jews as foreign workers’.36 Pevtchin duly asked for, and received, an ‘immediate’ operation to his nose.37

**Figure 4:**
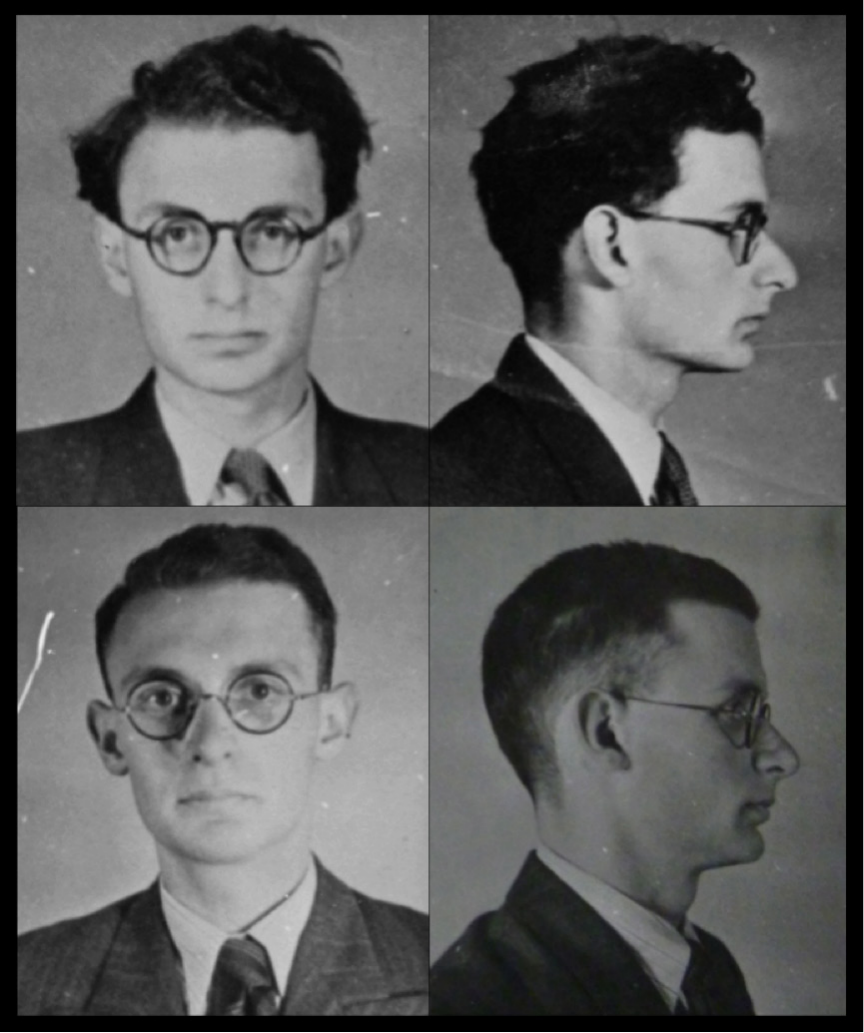
Peter Pertschuck photographed before (top) and after (below) surgery. Evidence of rhinoplasty can be clearly seen. TNA HS 7/49.

A second Jewish recruit who requested that procedure was Peter Pertshuck (Figure 4). Brought up in Paris and London, he was the younger brother of Maurice Pertshuck, an SOE agent arrested in France in 1943, and was serving in the Royal Air Force when he volunteered: SOE considered that ‘the fact that he [Peter] has had no news of his brother, whom he is very fond of, is probably the main reason for him joining this organisation’.38 As he proceeded through his training, SOE also noted that the younger Pertschuck wished ‘to undergo an operation to straighten his nose… he fears very much that his Jewish appearance might give him away’.39 That operation was carried out, after which he was described as ‘operationally ready’.40 Comparisons to photographs in his family’s hands confirm that Pertshuck was among the men whose before-and-after images illustrate the Camouflage Section’s history.41

Although the war ended before either could deploy, Pevtchin and Pertschuck’s readiness to alter their appearance offers a fresh perspective on interpretations of cosmetic surgery as a response to racial prejudice and cultural norms. Sander Gilman has explored how Jews have sought surgery to assist them in ‘passing’ as members of a more socially desirable group; within this context, he argues that ‘aesthetic’ surgery, as he terms it, is primarily about social stereotyping and feelings of unhappiness that precipitate transformation.42 Clearly Pevtchin and Pertschuk wished to appear less Jewish. With Pertschuk, SOE felt that surgery would prepare him psychologically, too: ‘considering his anxiety’ about his appearance, SOE recorded before it was done, it ‘would be a very advisable thing to do; it will give him this extra confidence which he needs’.43 Yet, neither man appears to have undergone surgery to ameliorate feelings of racial inferiority; indeed, both asked for and received it in the interests of subverting and destroying a society defined by its intolerance and persecution of the Jewish race. Acknowledging these drivers should qualify Foucauldian notions of biopower as a force for policing conformity.44

**Figure 5:**
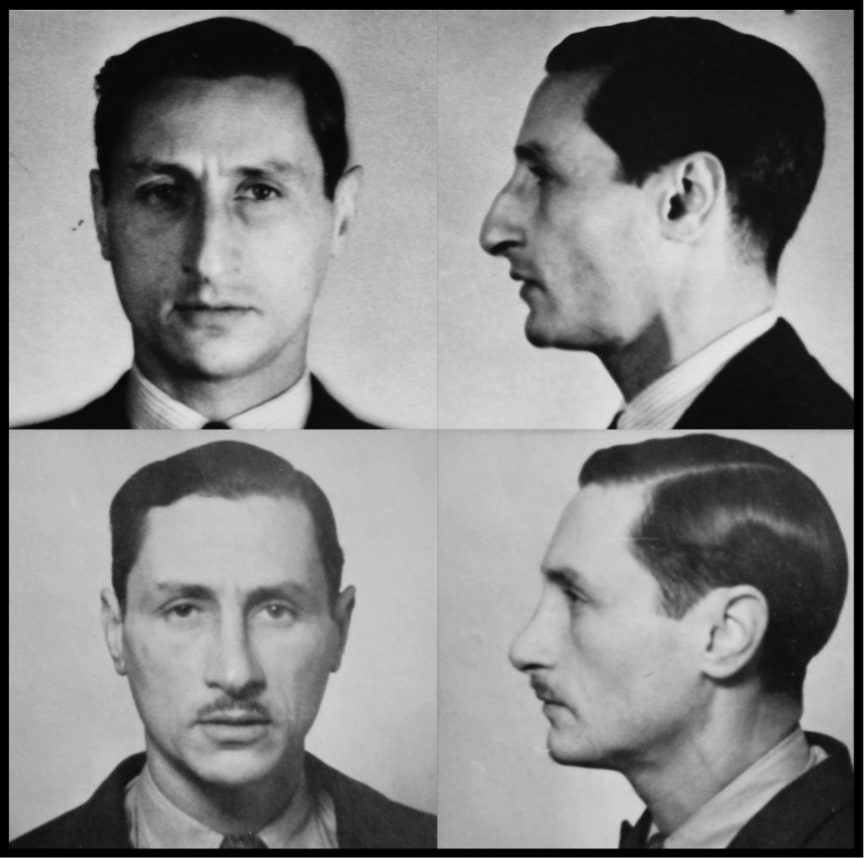
Frederick Lowenbach photographed before (top) and after (below) surgery, showing evidence of rhinoplasty. TNA HS 9/944.

Other motives were apparently at work when surgery was sought, however. Romanian-born but naturalised French, Frederick Lowenbach, the third Jewish agent who received surgery, had joined SOE in 1943 after earlier employment in France with Britain's Secret Intelligence Service (SIS). He underwent his operation shortly before returning to France in 1944 as an agent of SOE’s D/F Section, which specialised in running lines of communication into and out of occupied Europe. Later, recommending him for an award in recognition of his accomplishments in France, SOE noted that SIS had considered him ‘too compromised to be of any further use’: an obstacle, according to the citation, that Lowenbach had sought to circumvent by undergoing ‘a major facial operation in the hope that his appearance might be sufficiently changed to enable him to pass unnoticed’.45 Though details of the operation are not specified, photographs in his personnel file (Figure 5) show clear alterations to his nose.46 But Lowenbach’s desire for surgery was possibly more complicated than this. He had been ‘most anxious to have an operation performed on his nose in order to alter his appearance’, observed a senior SOE officer before he underwent it: ‘The reason for this is that he has already spent some time in the field, where he experienced difficulties’.47 But in 1945, the same senior officer, who had recruited Lowenbach in 1943 and considered him ‘an excellent agent and a dependable officer’, added his impression that Lowenbach’s personal experience of persecution, specifically as a refugee in France in 1940 and ‘as a Jew of Roumanian [sic] origin, without nationality or papers’, had made ‘a tremendously deep impression on him’. He was ‘ashamed of being a Jew’ and his ‘eagerness… to undergo a facial operation was in large measure due to the fact that he thought his altered appearance (would give)… him an Aryan look’, while ‘his chief desire as a reward for his services was to obtain British nationality which would enable him and his family to make a fresh start.’48

### DEVIL’S WORK?

The fact that surgery was out-sourced to civilian professionals suggests that SOE saw the practice as valuable. Today, enough documentation survives to confirm the identities of two of these surgeons. The postwar recollections of one of them provide an intriguing impression of how he perceived his wartime role and the justifications for it, at least insofar as he articulated them for public consumption in 1950. Underlining the importance of context when considering the rationales for interventions of this sort, his account prompts discussion, too, of ethical and legal implications of modifying bodies for state and wartime purposes.

Several surgeons feature in published accounts. Hutchison remembered his as ‘Mr Mowlem, who carried out his surgery at the London Clinic’.49 Langelaan’s was a ‘Mr. Norton, a civilian who had little to say’,50 while Levy’s was ‘a Bulgarian surgeon, Dr. Bankoff’.51 A fourth described in print as engaging in this work was Sir Harold Gillies, the most distinguished plastic surgeon in Britain at the outbreak of the Second World War. His biographer, Reginald Pound, mentions two occasions at Rooksdown House, a plastic surgery unit set up to treat army and civilian casualties, when Gillies operated on the faces of men apparently engaged in secret work. In a 5-hour operation on a subject of whose nationality he was unaware, Gillies altered the shape of an eyebrow, rounded the tip of the nose and slightly dilated the nostrils. The second operation was on a young Englishman. This time Gillies removed part of the chin, which he implanted in the patient’s hip so that it could be replaced if he survived the war. According to Pound, this man did not return for that procedure.52

Corroboration of Gillies’s involvement has still to surface from contemporary paperwork. No evidence of a wartime surgeon called ‘Mr Norton’ has come to light anywhere; probably that name was a pseudonym. But ‘Mr Mowlem’ and ‘Dr Bankoff’ do appear in SOE files.

The former was Rainsford Mowlem. Like Gillies, Mowlem was a New Zealander with an established career in Britain as a professional plastic surgeon. For much of the Second World War, he worked at Hill End Hospital in Hertfordshire, principally with injured servicemen.53 He also shared with Gillies a Harley Street practice close to the London Clinic, which was where James Hutchison would claim to have undergone his surgery. Today, the declassified file of one French agent confirms Mowlem’s connection to SOE. This agent was Henri Derringer, a 38-year-old cavalry officer brought out from France to Britain in 1943. ‘There is no question but that Mr Derringer’s scar can be improved’, Mowlem assured SOE in a surviving letter. ‘The chief advantage will be the elimination of surface irregularity as well as improvement in colour & texture. I will want him for a day and a night in the Clinic and then he can go and return for dressing’.54 How Mowlem’s services were enlisted remains unclear. Hutchison indicates that SOE’s chief medical consultant, a respected London physician, had helped secure Mowlem for his operation.55 That was months after Derringer’s proposed treatment, however. It was also long after an occasion when SOE sent a recruit to Mowlem’s hospital for secret treatment to a training injury.56

Less established, perhaps, was SOE’s connection to George Bankoff. Born in Bulgaria in 1903, he had worked in Italy and Germany before moving to Britain in the early 1930s and securing British nationality in 1938.57 Three years later, he came to SOE’s attention as a prospective agent, after apparently telling a friend that, if he could reach Switzerland, he ‘could arrange to become one of Hitler’s medical attendants’.58 ‘He is a highly qualified surgeon and was connected for a number of years with the Italian Hospital in London’, SOE noted.59 That venture was not pursued, due apparently to an MI5 report that Bankoff, at some unspecified time, had held ‘very strong pro-Italian sympathies’.60 When, 2 years later, Section XV engaged him as a plastic surgeon, that engagement, too, did not last long, due, again, to doubts about his background. ‘The work for which you propose to use him is extremely confidential’, an SOE security officer advised Section XV, ‘in that, if anyone describes [publicly] the nature of the operations performed, their whole object is lost… I think that this type of work should be performed by a man with entirely British outlook and I should have thought that there were some such surgeons available’.61 In 1946, J W Munn, wartime head of SOE training, recorded his belief that Bankoff had been employed long enough to handle two SOE matters: one was a surgical operation (of which no corroborating details have surfaced, though the timings fit with Jean-Pierre Levy’s stay in London); the other was a prescription for someone’s heart tablets.62 But it is likely that Bankoff did more than this. Flemming Muus did not name his surgeon, but the Imperial War Museum holds in its archives, among the private papers of the SOE representative who countersigned it, a consent form, signed by Muus, agreeing to surgery at Bankoff’s hands ‘the object of which is to alter my face’.63 Perhaps Munn had forgotten or never heard about some of Bankoff’s services. It is also conceivable that Bankoff—and, indeed, Gillies and Mowlem—had worked for other organisations that sent agents into enemy territory, like SIS.

Those sources also support Bankoff’s postwar claim to have carried out exactly this kind of wartime work. A prolific writer of popular fiction and fact, he would pen over 100 books during his lifetime. He also used a series of pseudonyms: his first book, *The Healing Knife*, a well-received account of his early career, was written as ‘George Sava’.64 It was as ‘George Borodin’ that he wrote *No Crown of Laurels*, published in 1950, which purported to be the memoir of a plastic surgeon who, during the war, had been employed to change agents’ appearances.65 The book described the stories of a dozen or so individuals, their backstories and their missions, and the bespoke techniques used on each one; for example: nose-shortening, by removing a piece of the septum; nose-narrowing, by removing cartilage from the sides of the nose; nose-broadening, by inserting extra cartilage between the skin and mucous membrane; face-lifting, to smooth cheeks and forehead creases; skin grafts, to erase or fabricate scars; making ears less prominent by pinning them back and removing skin and cartilage; and inserting fat to fill out fleshier parts of the face. The intelligence historian Nigel West has dismissed those stories as ‘bogus’ and Bankoff’s claims for his wartime career as ‘entirely spurious’.66 West argues that none of Bankoff’s tales can be independently corroborated and that he could not have learnt such intimate details about his subjects’ identities and secret exploits. But then, *No Crown of Laurels* does not claim to be accurate. Overlooked by West, an opening Publisher’s Note explains that ‘the author… actually operated on various Resistance leaders [but] has cast this story in a fictitious mould by giving imaginary names to the leading characters and by adapting events and circumstances’.67

*No Crown of Laurels* is the only published account of this practice from a surgeon’s point of view. It also presents its author as a shocked victim of circumstance, dismayed when the ‘War Office’ compelled him to do work conflicting with standard justifications of his craft.

Plastic surgery is an art, a science, a skill – call it what you like – which aims at restoring human beings to the semblance and functions of normal men and women when they have been ravaged by disease or accident or by the hand of Nature. It strives to make normal bodies out of abnormal ones, to turn ugliness into comeliness.

It was none of these things that I was being asked to do. My new task was something very different, a gross distortion that was horrible in all its implications. For I was to receive normal people and maim their faces and their limbs so that they were no longer the same people to look upon.

[I]t seemed then, as it was to seem through all the years of war, and does still seem in retrospect, a grinning mockery of all that surgery stands for.

Bankoff claimed that he had had to comply: it was ‘an order’. In later pages, he repeated his belief ‘that it was a debasement of surgery and a denial of the whole of the ethics of medicine. It was nothing but the *force majeur* of national necessity that had won my consent. I still feel that it was a prostitution of the science of healing’. Elsewhere in the book, however, he framed this work as valuable and ‘justified’: ‘in war, ethics do not count. It is the end not the means that matters’. He had gained satisfaction from contributing ‘to a greater cause, a finer effort, than most of the affairs of peace’ and dedicated his book ‘to those with whom I had the honour to be associated in a small degree’, praising ‘their undaunted courage and their great humanity which made them see themselves small and insignificant in comparison with the freedom and happiness of all men’.68

It is impossible to tell if Bankoff’s conflicted feelings, as expressed here, were genuine. He did not expand on what he meant by ‘ethics’, but little in the way of formal standards or regulations for cosmetic surgery had existed during the war: as one historian of bioethics has written, ‘there were no official British guides to good (ie, ethical) medical practice until the 1980s… Through the end of the twentieth century medical ethics in Britain tended to be conceptualized in terms of an ethos of honor and a discourse of “the done thing”’.69 Possibly Bankoff’s views were influenced by those of 1950, a time of growing awareness of principles like informed consent, though still long before the birth of bioethics as a field. It can also be noted that, both during and after the war, Bankoff was a man with books to sell and a living to make, a vested interest in promoting cosmetic surgery as a respectable enterprise, and, perhaps, a few other points to prove, given that the authorities had not always considered him trustworthy.

Grounds exist on which to interpret Bankoff’s unease as convincing, nevertheless. Describing himself as ‘the devil’s make-up man’, he had accurately identified a paradox that, by transforming bodies into forms able to work in enemy territory, surgery exposed them to conditions of heightened danger. ‘The preparation was mine [and] it meant opening the way to death rather than the sunlit path to life’.70 Possibly, its implications were more serious than this. Surgery, as OSS put it, made a ‘valuable’ man ‘less noticeable, harder to describe, more one of the crowd’.71 This deception was designed to preserve life; but it also risked making its recipients vulnerable, if they were captured and their true identities became known, to legal charges of perfidy and espionage.

According to the 1907 Hague Convention, which applied in the 1940s, it was ‘especially forbidden’ to ‘kill or wound treacherously individuals belonging to the hostile nation or army’: an acknowledgement of a general obligation that military forces had to fight wearing a form of uniform distinguishing themselves from civilians, and that donning civilian clothing with a clear intent to deceive—an act of perfidy—would violate the law of war if it was the proximate cause of killing or wounding.72

The same convention defined a spy as a person who, ‘when, acting clandestinely or on false pretenses obtains or endeavours to obtain information in the zone of operations of a belligerent, with the intention of communicating it to the hostile party’.73 Although espionage was not a war crime under international law, neither the Hague Convention nor the 1929 Geneva Convention, which applied in the 1940s to the treatment of prisoners, offered much protection to captives deemed to be spies, except for establishing that no one suspected of spying should be punished without first being tried.74 If a trial was fair, then severe punishment, if properly in line with the captor government’s domestic penalties for a proven offence of espionage, could legitimately follow.75 This was the fate, for example, of Emilio Zappala, who, when interrogated after capture, disclosed Giovanni Di Giunta’s surgery and secret mission. A clandestine agent of Britain’s SIS, Zappala had been arrested in Italian-controlled Sicily while wearing civilian clothes and carrying faked documents. Weeks later, he was put before a Fascist tribunal in Rome, found guilty of committing espionage, sentenced to death for that offence, and shot.76

So far, no agent who received surgery has been identified as being killed or captured, and no evidence suggests that SOE ever felt that modifying agents’ bodies might do them harm as well as good. It is pertinent to note, however, that 10 Australian and British commandos, captured near Singapore in 1944, were found guilty of perfidy and espionage by a Japanese military court—and subsequently executed—partly on the grounds of having used skin dye to look like Malay civilians.77

Also, while the act of enhancing bodies was not illegal, its potential to endanger the rights of those responsible could have extended to surgeons, too. As it applied in the Second World War, the Geneva Convention provided for the protection of medical personnel ‘engaged exclusively in the collection, transport and treatment of the wounded and sick, and in the administration of medical formations and establishments’.78 Surgeons who behaved otherwise, including, conceivably, transforming the bodies of healthy individuals into forms more effective at evading detection, risked undermining their special status as protected non-combatants.

SOE may not have gone as far in this direction as OSS, whose own files reveal how one American-run agent, sent into Germany disguised as a wounded German soldier travelling home from an army hospital, was provided with the medical means of keeping an existing arm injury unhealed, and even appear worse, to make his story more convincing; and how another, ‘desiring to pose as a German SS member’, received, beneath his armpit, the distinctive tattoo by which all SS soldiers recorded their blood type.79 But it remains the case that SOE’s use for facial surgery was not designed to treat physical ailments, restore appearance or counter feelings of mental distress, and, while ostensibly meant to safe life, may have exposed those involved, both recipients and surgeons, to elevated risks of significant harm. As such, and as a novel illustration of the lengths to which states have historically gone to deceive and defeat their enemies, it has relevance to current debates about the implications of similar practices in modern conflict. In bioethics scholar Eric Juengst’s much-quoted definition, ‘human enhancement’ is an intervention designed to ‘improve performance, appearance, or capability besides what is necessary to achieve, sustain or restore health’.80 In a military context, this can include, for example, biochemical developments, such as the use of pharmacological stimulants to counter fatigue, pain, and psychological trauma, and innovations in cybernetics and biomechanics.81 Although historians interested in military enhancement of the body have largely confined their attention to drugs,82 the perceived benefits of all of these developments include improved fighting capacity and reduced casualty rates.83 Their possibilities also provoke concern about ethics and legality.84 From that perspective, and considering the ability of plastic surgery to currently confound, for example, technologies of facial recognition,85 it may be instructive to reflect on the wartime work of surgeons who, at the state’s behest, fashioned bodies into more effective weapons by disguising their identities. A 1977 addition to the Geneva Conventions of 1949, which applies today, specifically deems ‘feigning civilian, non-combatant status’ to be perfidy, since it deliberately takes advantage of claims to protection,86 while at least one specialist in law and war has recently argued that supervising biomedical enhancements, such as drugs to augment the ability of ‘warfighters’ to withstand fatigue, threatens the protected status of medical personnel.87

## CONCLUSION

Despite the declassification of contemporary documentation, aspects of wartime surgery for facial disguise remain hard to penetrate. No figures or lists of recipients seem to survive among available records, which makes it impossible to estimate the extent to which surgery aided SOE’s efforts or, indeed, extended to camouflaging women, though Bankoff wrote of working on some. Nor do surviving records permit much assessment of its effectiveness. ‘Only once was I recognised’, Langelaan recalled. ‘I telephoned to an old friend who, hearing my voice without having seen my new face, knew me instantly’.88 ‘The result was pretty good’, Muus reflected on his own operation; ‘anyway, at a later date my mother did not recognise me when I passed her at a distance of twenty feet’.89 Jean-Pierre Levy was of the opinion that the removal of his scar ‘succeeded perfectly’, pointing out that his identity papers no longer had to mention it.90 But anecdotal testimony is all that exists; and it is not all positive. Muus’s wife would complain after the war that ‘they hadn’t done a very good job: they’d left some very big scars along his ears. Well, he was the only man in Denmark who had those scars, so he was terribly vulnerable to being recognised’.91 To judge from SOE reports, the tidying of Henri Derringer’s scar—if Mowlem’s treatment went ahead, which Derringer’s file does not confirm—may not have helped him much: dropped by parachute near Angers in 1943, ‘from the first he experienced difficulties. He was too well known in the district’. After 3 months he relocated to Paris.92

The ‘provider-user’ dynamic, as Thomas Schlich has described surgeon–patient relationships, also remains rather opaque.93 Pertshuck, Pevtchin and Lowenbach are recorded as requesting surgery. Langelaan and Hutchison recalled meeting their surgeons beforehand and discussing the work to be done.94 Otherwise, available sources—Flemming Muus’ consent form notwithstanding—say little about whether subjects trusted their surgeons, understood the techniques, were aware of the risks of an operation going wrong or the possible consequences of capture, or, indeed, consented to the procedures that they underwent. From the surgeon’s side, Bankoff’s concerns have been impossible to corroborate; it is unclear, too, whether his or Mowlem’s civilian status, which positioned them outside the hierarchical structures of obligation within which military surgeons usually worked, affected their participation in any way.

Also hard to illuminate is how recipients adjusted to their faces being permanently changed. The physiological, psychological and social challenges of facial surgery can be extremely significant, as Sharrona Pearl and Fay Bound Alberti have emphasised in the context of face transplants.95 Awareness and understanding of these effects are still in their fledgling phase, and further study of facial surgery for disguise, as an intervention performed for a short-term purpose unconnected to beauty or the restoration of physical function, might provide intriguing insights. Peter Pertschuk’s family remembers that his voice was slightly affected96 but no other long-term consequences have so far been unearthed, except, perhaps, the fact that George Langelaan, one of the first to receive this surgery, later penned the body-transformation/horror story, *The Fly*.97

Enough detail exists, however, to enhance understanding not only of this unique purpose for plastic surgery but also of conflicting and contrasting perceptions of it. This picture offers a fresh measure of lengths to which states have gone to confront their enemies and could go again: for SOE, these procedures were a technical and acceptable solution to the problem of ‘personal camouflage’. It is also a snapshot of constructed ideas of difference around the body and the complex motives of those who have wished to alter it. Underlining Pearl’s call to consider ‘the subjective experience’ of facial surgery and ‘the link between a specific face and an individual character’, Frederick Lowenbach’s apparent desire for the procedure, for example, presents a striking illustration of surgery as a response to concerns that facial features were distinguishing markers of race and a hindrance socially.98 Studying perceptions of these interventions underlines, too, the importance of considering the relevance of time and place. In a monograph published in 1943, 7 years before proclaiming, in peacetime, that applying his skills to disguise bodies had been ethically problematic, George Bankoff had described cosmetic surgery as ‘a social necessity imposed by the conditions of the times’: an effective illustration of how completely rationales for it can change according to context.99

## Notes

1. Records of the Livorno Questura, (Ministero dell’Interno, Direzione Generale Della Pubblica Sicurezza) to multiple recipients, ‘Oggetto: Di Giunta Giovanni’, 17 November 1942, Records of the Livorno Questura, Archivio di Stato, Livorno, Italy.

2. Major J. Pearson to Major C. Roseberry, 5 August 1942 and 16 September 1942, TNA HS 6/821.

3. SOE War Diary, May 1942, TNA HS7/232.

4. Roderick Bailey (2015), *Target Italy: The Secret War against Mussolini, 1940-1943. The Official History of SOE Operations in Fascist Italy* (London: Faber & Faber, 2015: paperback edition), 362-3.

5. Flemming B Muus (1956), *The Spark and the Flame* (London: Museum Press Limited, 1956); George Langelaan (1959a), *Knights of the Floating Silk* (London: Quality Book Club, 1959); Sir James Hutchison (1977), *That Drug Danger* (Montrose: Standard Press, 1977); Jean-Pierre Levy (1998), *Mémoires d'un franc-tireur: Itinéraire d'un résistant* (1940-1944) (Paris: Complexe, 1998).

6. Roderick Bailey (2014), “Secret Scalpel: Plastic surgery for wartime disguise,” *Lancet* 384, (2014): 1421-2.

7. Sharrona Pearl (2017), *Face/On: Face Transplants and the Ethics of the Other* (Chicago: University of Chicago Press, 2017), 26, 33, 150-3.

8. Sander Gilman (1991), *The Jew's Body* (New York: Routledge. 1991); Sander Gilman (1998), *Creating Beauty to Cure the Soul: Race and Psychology in the Shaping of Aesthetic Surgery* (Durham, N.C.: Duke University Press, 1998).

9. Elizabeth Haiken (1997), *Venus Envy: A History of Cosmetic Surgery* (Baltimore: Johns Hopkins University Press, 1997).

10. Sharrona Pearl, *Face/On*.

11. Elizabeth Haiken, *Venus Envy*; Sander Gilman (1999), *Making the Body Beautiful: A Cultural History of Aesthetic Surgery* (Princeton: Princeton University Press, 1999).

12. Abeer Khalid and Carlos Quiñonez (2015), “Straight White Teeth as a Social Prerogative,” *Sociology of Health and Illness* 54, No.5 (2015): 786-8.

13. Thomas Schlich (2018), “Introduction: What is Special About the History of Surgery?” in *The Palgrave Handbook of the History of Surgery*, ed. Thomas Schlich (London: Palgrave Macmillan, 2018): 16.

14. Anon (1945). “Colonel’s Change of Face,” *Belfast News-letter*, 2 June 1945.

15. Air Commodore Archie Boyle to Commander John Senter, 2 June 1945, TNA HS 9/770/6.

16. Sir James Hutchison, *That Drug Danger*, 99-102.

17. Flemming B Muus (1950), *Ingen tænder et lys* (Copenhagen: Hanning Branners, 1950).

18. Flemming B. Muus, *The Spark and the Flame*, 51.

19. George Langelaan (1950), *Un Nommé Langdon* (Paris: Robert Laffont, 1950).

20. George Langelaan, *Knights of the Floating Silk*, 83-88. An American edition appeared as *The Masks of War: From Dunkirk to D-Day: The Masquerades of a British Intelligence Agent* (New York: Doubleday, 1959).

21. Jean-Pierre Levy, *Mémoires d'un franc-tireur*, 107 (author’s translation from French).

22. Thomas Schlich and Christopher Crenner (2017), “Technological Change in Surgery: An Introductory Essay,” in *Technological Change in Modern Surgery: Historical Perspectives in Innovation*, ed. Thomas Schlich and Christopher Crenner Parens (Rochester, N.Y.: University of Rochester Press, 2017), 1.

23. “History and Development of the Camouflage Section, 1941-1945,” 1945, TNA HS 7/49.

24. Ibid.

25. Lovell to Whitney Shephardson, 8 July 1943, US National Archives and Records Administration (hereafter NARA) Record Group (hereafter RG) 226, Entry 134, Box 52, Folder 567.

26. “Personal Disguise,” December 1944, NARA RG 226, Entry 210, Box 81.

27. “Progress report no. 22,” May 1943, and “Monthly report, Camouflage Section,” August 1943, NARA RG 226, Entry 134, Box 49, Folder 506.

28. “History and Development of the Camouflage Section, 1941-1945,” 1945, TNA HS 7/49.

29. Frederic Boyce and Douglas Everett (2003), *SOE: The Scientific Secrets* (Stroud: Sutton Publishing, 2003), 93.

30. Records of the Office of Strategic Services (Record Group 226) (2017). “British SOE Camouflage Sections XV and XVa,” 30 October 1944, NARA RG 226, Entry 99, Box 14, Folder 1.

31. Sir James Hutchison, *That Drug Danger*, 99.

32. Flemming Muus, *The Spark and the Flame*, 50.

33. George Langelaan, *Knights of the Floating Silk*, 82.

34. Arthur Kellas (1990), *Down to Earth* (Edinburgh: Pentland Press, 1990), 146-7.

35. “Form T.1: History Sheet,” 1943, TNA HS 9/1179.

36. Telegram, London to Brussels, January 1945, TNA HS 9/1179.

37. Telegram, Brussels to London, January 1945, TNA HS 9/1179.

38. Training school report, 12 April 1944, TNA HS 9/1172/8.

39. Training school reports, 12 and 25 April 1944, TNA HS 9/1172/8.

40. Application for Commission, 30 June 1944, TNA HS 9/1172/8.

41. Information from Anne Whiteside.

42. Sander Gilman, *Making the Body Beautiful:* 21-6, 124-44.

43. Training school report, 25 April 1944, TNA HS 9/1172/8.

44. Abeer Khalid and Carlos Quiñonez, “Straight White Teeth as a Social Prerogative”: 789-90.

45. Recommendations for the award of the OBE to Major F.C. Lowenbach, TNA WO 373/97.

46. Photographs, TNA HS 9/944.

47. Colonel Leslie Humphries to Brigadier Eric Mockler-Ferryman, 28 December 1943, TNA HS 9/944.

48. Note by Colonel Leslie Humphries, 14 April 1945, TNA HS 9/944.

49. Sir James Hutchison, *That Drug Danger*, 100.

50. George Langelaan, *Knights of the Floating Silk*, 81.

51. Jean-Pierre Levy, *Mémoires d'un franc-tireur*, 107.

52. Reginald Pound (1964), *Gillies: Surgeon Extraordinary* (London: Michael Joseph, 1964), 141-2.

53. Richard W Griffiths (2013), “Arthur Rainsford Mowlem,” *Journal of Medical Biography* 21, no.3 (2013): 184.

54. Rainsford Mowlem to Dr Isaac Jones, 29 July 1943, TNA HS 9/420/8.

55. Sir James Hutchison, *That Drug Danger*, 100.

56. Major Hamish Ireland to Mr. Mansfield, 16 August 1944, TNA HS 9/903/6.

57. Metropolitan Police report, 12 April 1938, TNA HO 405/2071; Certificate of Naturalization, December 1938, TNA HO 334/151.

58. Undated note by Section V, 1941, TNA HS 9/84/3.

59. Note, 3 May 1941, TNA HS 9/84/3.

60. “Remarks by MI5,” 3 May 1941, TNA HS 9/84/3.

61. Squadron Leader Hugh Park to Lieutenant Colonel J. Edgar Wills, 25 June 1943, TNA HS 9/84/3.

62. Colonel J. W. Munn to Major Norman Mott, 30 April 1946, TNA HS 9/84/3.

63. Consent form, 15 January 1943, Papers of Reginald Spink, Imperial War Museum, London, UK.

64. George Sava (1938), *The Healing Knife: A Surgeon’s Destiny* (London: Faber & Faber, 1938).

65. George Borodin (1950), *No Crown of Laurels* (London: Werner Laurie, 1950).

66. Spink, Reginald (), *Counterfeit Spies* (London: Little, Brown, 1998), 7.

67. George Borodin, *No Crown of Laurels*, 5.

68. Ibid., 11-12, 21, 161, 205.

69. Robert B Baker (2009), “The Discourses of Practitioners in Nineteenth- and Twentieth-Century Britain and the United States,” in *The Cambridge World History of Medical Ethics*, ed. Robert B Baker and Laurence B McCullough (Cambridge: CUP, 2009), 460, 451, 461.

70. George Borodin, *No Crown of Laurels*, 12.

71. Newton Jones to Major Watts Hill, 10 October 1944, NARA RG 226, Entry 134, Box 52, Folder 567.

72. Hague Convention IV (1907) The Laws and Customs of War on Land, Article 23 (1907); W. Hays Parks (2003), “Special Forces’ Wear of Non-Standard Uniforms,” *Chicago Journal of International Law* 4, no.2 (2003): 521-2, 541-2.

73. Hague Convention IV (1907): The Laws and Customs of War on Land, Article 29 (1907).

74. W. Hays Parks, “Special Forces’ Wear of Non-Standard Uniforms”: 512.

75. Ibid: 544.

76. Roderick Bailey, *Target: Italy*, 148-151.

77. ‘Translation of Proceedings of Military Court of 7th. Area Army’, 1945, TNA HS 9/775/4.

78. Geneva Convention on Prisoners of War, Article 9 (1929).

79. “Cover and Authentication Branch: Field Manual – Strategic Services,” 1945, NARA RG 226, Entry 210, Box 330, Folder 13091.

80. Eric Juengst (1998), “The Meaning of Enhancement,” in *Enhancing Human Traits: Ethical and Social Implications*, ed. Erik Parens (Washington DC: Georgetown University Press, 1998), 29.

81. Harrison Dinniss (2016), “Soldier 2.0: Military Human Enhancement and International Law,” *International Law Studies* 92, (2016): 434-6.

82. Łukasz Kamieński (2015), *Shooting Up: A History of Drugs and War* (Oxford: OUP, 2015); Norman Ohler (2016), *Blitzed: Drugs in the Third Reich* (London: Allen Lane, 2016).

83. Matthew Beard, Jai Galliott, and Sandra Lynch (2016), “Soldier Enhancement: Ethical Risks and Opportunities,” *Australian Army Journal* 8, no.1 (2016): 6-13.

84. Jai Galliott and Mianna Lotz (2015)., *Super Soldiers: The Ethical, Legal and Social Implications* (Farnham: Ashgate, 2015).

85. Michele Nappi, Stefano Ricciardi, and Massimo Tistarelli (2016), “Deceiving Faces: When Plastic Surgery Challenges Face Recognition,” *Image and Vision Computing* 54 (2016): 71-82.

86. Additional Protocol to the Geneva Conventions of 12 August 1949, Article 37(1) (8 June 1977).

87. Rain Liivoja (2018), “Biomedical Enhancement of Warfighters and the Legal Protection of Military Medical Personnel in Armed Conflict,” *Medical Law Review* 26, no.3 (2018): 421-448.

88. George Langelaan, *Knights of the Floating Silk*, 88.

89. Flemming B. Muus, *The Spark and the Flame*, 51.

90. Jean-Pierre Levy, *Mémoires d'un franc-tireur*, 107.

91. Varinka Muus (1994), Sound Archive interview no. 13104, Imperial War Museum, London.

92. “Interrogation of Toinot @ Gerard,” 11 April 1944, TNA HS 9/420/8.

93. Thomas Schlich, “Introduction: What is Special About the History of Surgery?”: 8.

94. George Langelaan, *Knights of the Floating Silk*, 81-3; Sir James Hutchison, *That Drug Danger*, 100.

95. Sharrona Pearl. *Face/On*; Fay Bound Alberti, “From Face/Off to the Face Race: The Case of Isabelle Dinoire and the Future of the Face Transplant,” *Medical Humanities* 43 (2017): 148-54.

96. Information from Anne Whiteside.

97. George Langelaan (June 1957), “The Fly,” *Playboy* (1957).

98. Sharrona Pearl, *Face/On*, 24-5.

99. George Bankoff (1943), *Plastic Surgery* (London: Medical Publications, 1943), 10, 12.
